# The genomics of insecticide resistance: insights from recent studies in African malaria vectors

**DOI:** 10.1016/j.cois.2018.05.017

**Published:** 2018-06

**Authors:** Chris S Clarkson, Helen J Temple, Alistair Miles

**Affiliations:** 1 Wellcome Sanger Institute, Hinxton CB10 1SA, United Kingdom; 2 The Biodiversity Consultancy Ltd, Cambridge CB2 1SJ, United Kingdom; 3 Big Data Institute, Oxford OX3 7FZ, United Kingdom

## Abstract

Over 80% of the world's population is at risk from arthropod-vectored diseases, and arthropod crop pests are a significant threat to food security. Insecticides are our front-line response for controlling these disease vectors and pests, and consequently the increasing prevalence of insecticide resistance is of global concern. Here we provide a brief overview of how genomics can be used to implement effective insecticide resistance management (IRM), with a focus on recent advances in the study of *Anopheles gambiae*, the major vector of malaria in Africa. These advances unlock the potential for a predictive form of IRM, allowing tractable feedback for stakeholders, where the latest field data and well parameterised models can maximise the lifetime and effectiveness of available insecticides.

## Highlights

• 80% of the world’s population is at risk from arthropod-vectored diseases.
• 450 million clinical cases of malaria were averted via insecticide treated bednets.
• Some populations of malaria mosquitoes are resistant to multiple insecticide classes.
• Genomics reveals how insecticide resistance evolves and spreads.
• Genomics can facilitate adaptive management of future vector control campaigns.

**Current Opinion in Insect Science** 2018, **27**:111–115

This review comes from a themed issue on **Pests and resistance**

Edited by **Christopher Jones** and **Chris Bass**

For a complete overview see the Issue and the Editorial

Available online 05 June 2018

**https://doi.org/10.1016/j.cois.2018.05.017**

2214-5745/© 2018 The Authors. Published by Elsevier Inc. This is an open access article under the CC BY license (http://creativecommons.org/licenses/by/4.0/).

## Introduction

Almost 82% of the world’s population is at risk from at least one arthropod-vectored disease, accounting for over 10% of the global disease burden [1]. Crop pests also exact a heavy toll, costing global agriculture an estimated $540 billion each year [2]. Insecticides remain the mainstay of efforts to control these disease vectors and pests, and are thus vital for preventing illness and maintaining food security. Of all vector-borne disease, malaria has the greatest impact on human health [3,4], and provides an example of the enormous potential benefit of public health programmes of vector control. Since the turn of the millennium there has been a massive scale-up of malaria vector control in Africa, primarily using insecticide-treated bed-nets (ITNs) and programmes of indoor residual spraying (IRS), which together account for 81% of the 663 million clinical malaria cases that are estimated to have been averted from 2000 to 2015 [5^••^]. Since 2015, however, further reductions in malaria prevalence appear to have slowed or stalled in some African regions [3], and there is growing concern over the rise of insecticide resistance (IR) [6^••^].

By 2012, approximately 54% of ‘at risk’ African households owned at least one pyrethroid impregnated ITN. Coverage of IRS programmes has also expanded greatly, with nearly two thirds of programmes using pyrethroids [6^••^]. Pyrethroids were also introduced into agriculture in Africa prior to the scale-up of public health vector control programmes, and continue to be used on a variety of crops such as cotton [7]. Mosquito populations have thus been bombarded with pyrethroids for nearly two decades, and it is now rare to find a malaria vector population in Africa without some degree of pyrethroid resistance [6^••^]. Pyrethroids are still the only insecticide class approved for use in ITNs, however four other insecticide classes are now available for use in both IRS and agriculture. Resistance to these other insecticide classes is less common among malaria vectors, but there are populations where exposure has resulted in resistance to multiple insecticide classes [8]. There is an ongoing debate regarding the epidemiological impact of IR, but the spread of resistance is alarming and there is a broad consensus that action must be taken [4,9^••^]. In practice, however, implementation of strategies for insecticide resistance management (IRM) remains a major challenge, for a number of reasons. These reasons include a lack of information regarding the molecular mechanisms of resistance, and regarding the geographical distribution and spread of resistance [9^••^]. Without this information, it is difficult to make informed decisions about optimal IRM strategies in a given location, or to formulate a coordinated response across larger regions.

Although the genetics of IR have been studied for more than six decades, recent advances in sequencing technology have brought about a revolution in our knowledge of the genetic basis of IR in *Anopheles gambiae*, the major vector of malaria in Africa. The construction of a high quality reference genome for *A. gambiae* in 2002 [10], just a year after the first public draft of the human reference genome [11], was a major leap forward, making possible a range of new techniques for large-scale, high-throughput discovery of IR-associated genes [12^••^]. In the last decade, a dramatic reduction in the cost of sequencing technology has meant that whole-genome sequencing (WGS) of thousands of mosquitoes collected from natural populations has become feasible, providing a further step-change in the quantity and richness of data available. In this paper we review how WGS is transforming the study of IR in African malaria vectors. We discuss how developments in genomics, together with related technologies and supporting tools, may provide a platform for vector population surveillance and enable predictions to be made about the response of a vector population to a given control intervention or IRM strategy. These developments should improve our capability to make informed choices that maximise the lifetime and effectiveness of available insecticides.

## New insights into the molecular basis of insecticide resistance

WGS studies are generating a wealth of new data regarding the molecular basis of IR, even for the most well-studied of genes. For example, the voltage-gated sodium channel (VGSC) is the molecular target of both DDT and pyrethroid insecticides, and variations within the amino acid sequence have been found to cause resistance across more than 40 species of insect [13,14]. Prior to WGS, this gene had been studied in *A. gambiae* via targeted capillary sequencing of specific exons and introns, leading to the discovery of two primary resistance variants (L1014F [15], L1014S [16]) and one secondary variant that substantially enhanced the resistance phenotype of L1014F [17,18]. Subsequently, the *Anopheles gambiae* 1000 Genomes Consortium (Ag1000G) undertook the first large-scale project using WGS to study natural mosquito populations, sequencing mosquitoes across a broad geographical range. The first phase of the Ag1000G project sequenced the genomes of 765 mosquitoes from 8 African countries, and discovered a total of 47 protein-altering mutations within the *Vgsc* gene, of which 17 were at appreciable frequency in one or more populations and appeared to be under selection [19^••^]. Some of these variants had previously been found to be associated with pyrethroid resistance in other insect species, but many were completely novel.

*Vgsc* is but one of many genes that have been associated with IR, and prior to WGS studies, even less was known about the genetic variation causing IR in these other genes. For example, the *Ace-1* gene is the target of carbamate and organophosphate insecticides, and a G119S substitution has been found to cause resistance to these insecticides in *A. gambiae* populations [20,21]. Studies combining WGS with other genetic methods have discovered that, in addition to amino acid substitutions, there are also large copy number variations spanning the *Ace-1* gene in the genomes of some mosquitoes, which provide another mechanism for IR via increased gene expression and/or permanent heterozygosis allowing escape from the fitness costs of carrying resistance mutations in the absence of insecticides [22]. Various genes have been associated with metabolic resistance to pyrethroids, but prior to WGS studies, the only known genetic marker of metabolic IR was a point mutation in the *Gste2* gene [23]. No genetic markers were known for metabolic resistance mediated by cytochrome P450 genes, despite the fact that these have repeatedly been associated with high levels of resistance in field populations [24,25]. The *Anopheles gambiae* 1000 Genomes Project has generated data on nucleotide polymorphism covering more than 90% of protein coding sequence in the *A. gambiae* genome [19^••^]. This includes genome regions containing metabolic IR genes, such as the *Gste* gene cluster and the *Cyp6p* gene cluster, providing a wealth of new genetic markers that can be used to detect metabolic resistance and investigate the molecular changes involved.

WGS data also provides a new route to the discovery of IR genes, via genome-wide scans for signals of recent selection (GWSS), and via genome-wide association studies (GWAS) [26,27,12^••^]. Although the genes encoding the binding targets of currently used insecticides are well known, there remains much uncertainty about which genes are responsible for metabolism of which insecticides, and even less is known about the role of other types of gene such as membrane transporters [12^••^]. Undoubtedly many new IR genes remain to be discovered, and this remains a critical priority, because discovery of IR genes can lead to new avenues for the design of insecticides and synergists. The use of WGS data in selection scans and association studies in *Anopheles* is still at an early stage, but results from Ag1000G phase 1 provide some indication of the potential value. Strong signals of selection were found in multiple populations at the *Vgsc*, *Gste* and *Cyp6p* loci, confirming the presence of genes playing a key role in the ongoing adaptive response to insecticide use across a broad geographical range [19^••^]. Similarly strong signals of selection were also evident at a number of genome locations that have not previously been associated with IR. Work is ongoing within the Ag1000G Consortium to identify the genes at these novel loci that are the most likely target of selection, and to investigate their potential role in IR.

## New approaches to vector population surveillance

Currently the IR profile of malaria vector populations in Africa is monitored by phenotypic assays, and by genotyping a handful of known resistance variants using genetic assays. This information is valuable, but across much of Africa where resistance to one or more insecticides is present [6^••^], it provides very limited information about the underlying mechanisms of resistance. It also provides almost no information at all about how resistance is spreading between different mosquito populations. WGS data enable analyses not only of variation within IR genes, but also non-coding variation within introns and gene flanking regions. Combined with the application of statistical methods that estimate haplotypes from diploid genotypes at SNPs across the whole genome, these data enable an analysis of the genetic backgrounds carrying resistance alleles. If the same resistance allele is found on the same genetic background in mosquitoes collected from two different locations, then we can infer that the resistance allele has spread to those two locations from a common origin. Conversely, if the same allele is found on different genetic backgrounds, that implies independent local origins of resistance. When these analyses were applied to data from Ag1000G, it was possible to identify a number of distinct genetic backgrounds carrying important resistance alleles, and to show that some of these backgrounds are geographically localised whereas others have spread over thousands of kilometres [19^••^].

**Figure 1:**
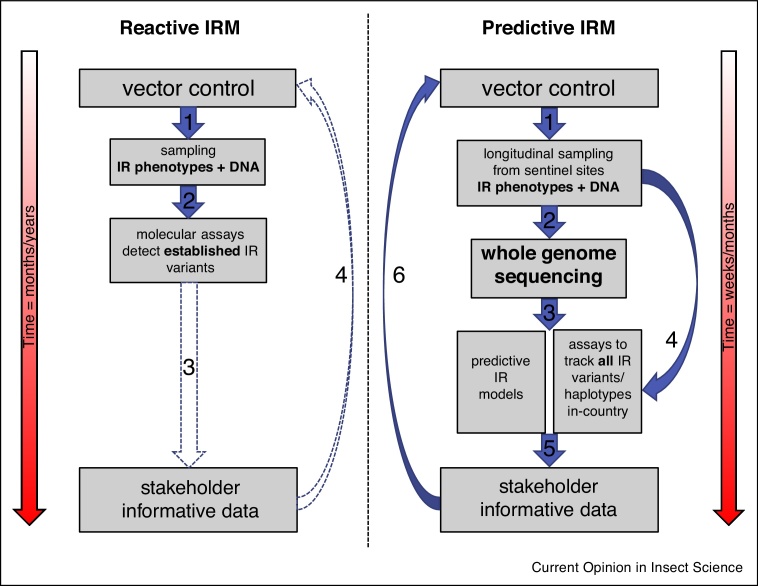
Insecticide resistance management flow diagram. Reactive IRM** — **an example IRM work flow without an active genomic component. **1.** Insects are sampled from a region undergoing a vector control campaign, these samples can be subjected to a bioassay to determine their IR phenotype after which their DNA is collected. **2.** Molecular assays for a small number of previously established IR associated genetic loci are conducted on the DNA to determine potential causal genotypes of the IR phenotype, to genetically characterise the population, this can help determine the mode of resistance, for example, target site/metabolic. **3.** This can provide useful information about insecticide resistance, but the speed with which it can be passed to vector programme managers in a readily useable format may be delayed by processing time and by the fact that these assays are often conducted outside the country of collection. **4.** Input from molecular assays can be used to improve IRM, but two key limitations mean that this approach is unlikely to be sufficient to fully prevent insecticide resistance, turnaround is too slow (months/years) and only established IR variants can be detected. Predictive IRM** — **an example IRM work flow with an active genomic component. **1.** Insects are sampled from a region undergoing a vector control campaign, representative ‘sentinel’ sites within the region are sampled repeatedly over time. Ideally the initial time points are taken before the IRM is rolled out. These samples can be subjected to a bioassay to determine their IR phenotype after which DNA is collected. **2.** DNA is sequenced in the country of collection and this, in concert, with advances in sequencing speed, reduces the time taken to generate data. **3.** Data produced from longitudinal sampling and whole genome sequencing can be used to parameterise predictive IR models and GWAS/GWSS can locate novel IR loci allowing molecular assays to cover all potential IR linked variants/haplotypes in natural populations. **4.** With strategic whole genome sequencing to update assays and model parameters, most samples can then skip the WGS step and be quickly and cheaply assayed for IR linked loci in-country with minimal technological requirements. **5.** As all data is already within the country of collection, it can be quickly passed to national/local vector control program managers. **6.** Using input from predictive models and genotype/phenotype associations, IRM can be modified rapidly enough (weeks/months) to avert the emergence and spread of insecticide resistance. The effectiveness of these modifications is monitored as the cycle begins again, and further improvements to IRM can be made as needed.

With further sequencing of vector populations across the geographical range of the species, and with further developments in statistical methods for inferring demographic and genealogical histories from genomic data, it should be possible to locate the geographical origins of IR and reconstruct their transmission paths, in a way that is analogous to the analysis of infectious disease outbreaks. All of these features will be useful for informing IR control programmes through a deeper understanding of how IR evolves in natural mosquito populations. Longitudinal sampling in concert with WGS, spanning times before, during and after vector control interventions such as ITN campaigns would also allow evaluation of the demographic impact caused by the intervention [9^••^]. By inferring vector effective population size change (*Ne*), and by measuring IR allele frequencies and species composition over time, all possible from WGS data [19^••^], the impacts of interventions could be quantified and vector control improved (Figure 1).

## Towards a predictive approach to insecticide resistance management

The analyses described above can provide a wealth of retrospective information regarding demographic and evolutionary changes in vector populations. This information is valuable and allows reactive IRM, but if IR has already taken hold in a vector population it may be too late to enable an effective response. Furthermore, it may be of little or no utility in populations where no previous sampling has occurred. To move towards a predictive approach to IRM that can be used in any context, we suggest a threefold approach. First, we need to further reduce the cost of sequencing so that it can be deployed at scale, and continue technological advances that allow a faster turnaround of stakeholder-useful IR information. For example, Oxford Nanopore’s Minion allows not only long-read sequencing but also allows the actual sequencing to take place *in situ* with minimal requirements other than an internet connection and a basic molecular lab [28] (Figure 1). These advances mean that genomic data can be generated in the regions where the insects are collected, in hours or days, as was demonstrated during the recent Ebola and Zika virus outbreaks where the technology was used for fast disease diagnosis [29,30]. Second, computational pipelines for managing very large datasets must be improved and made easily accessible, leveraging availability of cloud computing infrastructure to reduce the time taken and knowledge required to process, prepare and analyse data. Third, we need to develop accurate, well-parameterized computational models for how resistance evolves under different insecticides and IRM strategies, and for how resistance spreads between mosquito populations (e.g. [31,32]), to enable accurate predictions and rapid responses (Figure 1). A clear parallel in the scope for the practical application of the genomics of resistance can be found in the advances in understanding drug resistance in the malaria parasite *Plasmodium falciparum*, where drug resistance threatens to render all existing treatment ineffective. By linking drug resistance genotype to phenotype accurately and rapidly using genomics, reporting of the drug resistance landscape to stakeholders is enabling dynamic tailoring of the anti-malarial drugs used to help prevent emerging resistance [33^•^].

## Conclusions

Within the next 5–10 years, it is feasible to anticipate that dynamic adaptive management techniques analogous to those used to tackle virus outbreaks and emerging drug resistance in malaria parasites could be rolled out to address the emergence of IR in major malaria vector species globally. This could provide a model for controlling the emergence and spread of insecticide resistance in other arthropods that currently exact a heavy toll on human health, food security, and economic development. Many reference genomes and other genomics resources are already available for these arthropods: 40 insect vector references (gene sets and other useful data) can be downloaded or explored online using VectorBase [34], and many other pest insects are being sequenced as part of the i5k Insect Genomes Project [35]. Together with advancing theory and techniques, these resources will allow researchers to investigate demographics, discover the genetic drivers of IR, and to develop assays to quickly, cheaply and accurately inform control campaigns. These kinds of dynamic approaches to managing pests and pathogens will be increasingly necessary to support human wellbeing and sustainable economic development in a rapidly changing world. In the same way that emerging resistance to antibiotic drugs is threatening many of the medical advances of the 20th and 21st centuries, IR is a complex and multifaceted challenge that will require scientists, policymakers, and practitioners to work together.

## Conflict of interest statement

Nothing declared.

## References and recommended reading

Papers of particular interest, published within the period of review, have been highlighted as:

• of special interest
•• of outstanding interest
